# Supplementary material for: Increased glycoprotein hormone yield in stably transfected CHO cells using human serum albumin signal peptide for beta-chains
Source: PeerJ. 2025 Feb 14;13:e18908. doi: 10.7717/peerj.18908 (PMC11831970; doi:10.7717/peerj.18908)
Supplement: Supplemental Information 3 [file peerj-13-18908-s003.docx]

CG A-chain
Loading order: MW ladder, standard, standard 95°C, NSP-CG, HSA-CG, Azu-CG, aSP-CG, NSP-CG 95°C, HSA-CG 95°C, Azu-CG 95°C, aSP-CG 95°C.


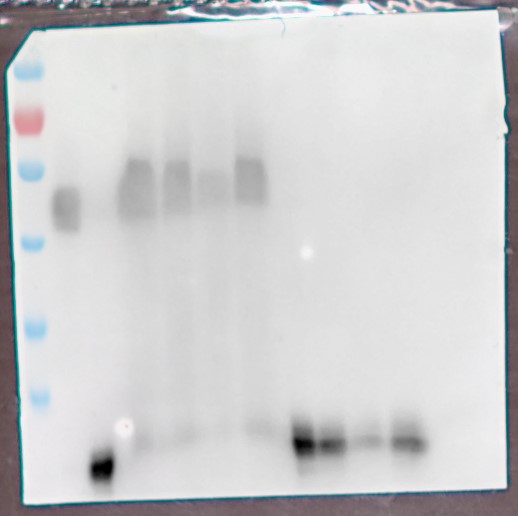


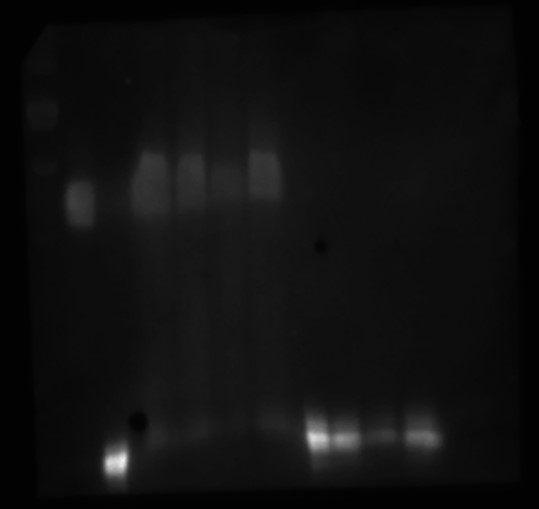


LH A-chain
Loading order: MW ladder, standard, standard 95°C, NSP-LH, HSA-LH, Azu-LH, aSP-LH, NSP-LH 95°C, HSA-LH 95°C, Azu-LH 95°C, aSP-LH 95°C, х, NSP-LH prec, HSA-LH prec, Azu-LH prec, aSP-LH prec.


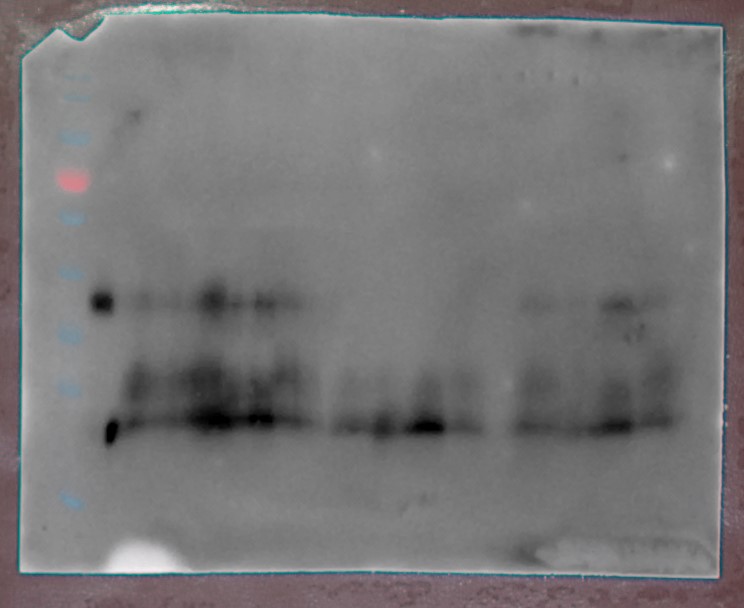


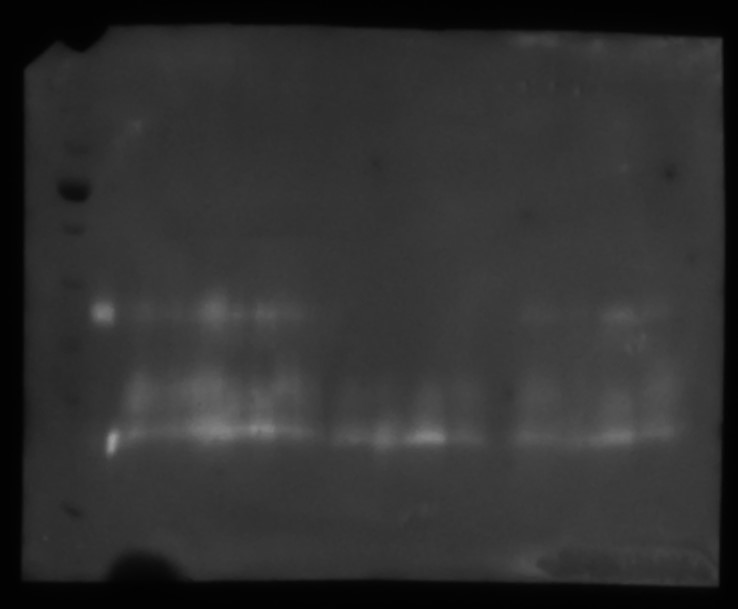


TSH A-chain
Loading order: MW ladder, standard, standard 95°C, NSP-TSH, HSA-TSH, Azu-TSH, aSP-TSH, NSP-TSH 95°C, HSA-TSH 95°C, Azu-TSH 95°C, aSP-TSH 95°C.


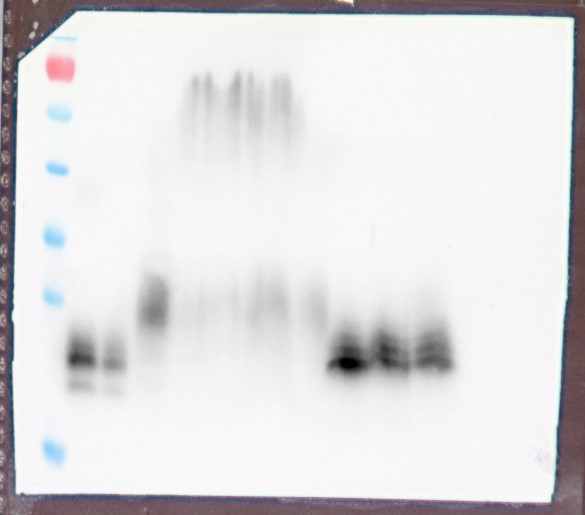


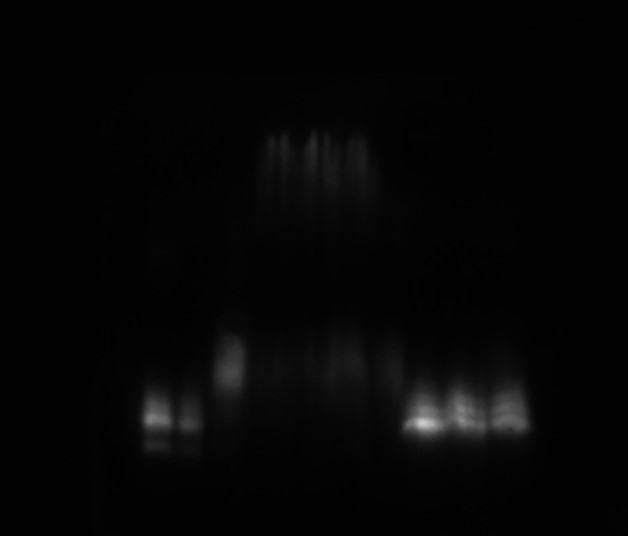


FSH A-chain
Loading order: MW ladder, standard, standard 95°C, NSP-FSH, HSA-FSH, Azu-FSH, aSP-FSH, NSP-FSH 95°C, HSA-FSH 95°C, Azu-FSH 95°C, aSP-FSH 95°C.


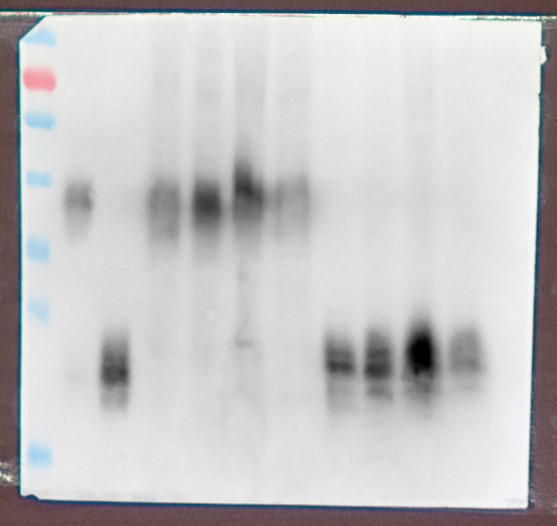


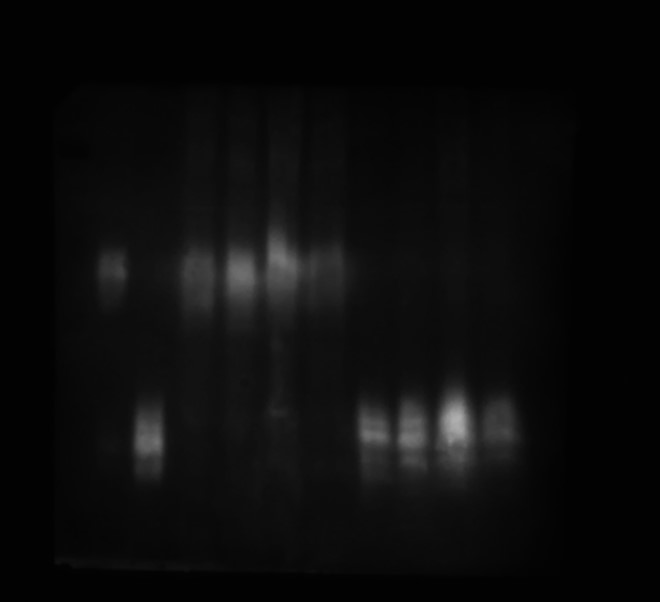


CG B-chain
Loading order: MW ladder, standard, standard 95°C, NSP-CG, HSA-CG, Azu-CG, aSP-CG, NSP-CG 95°C, HSA-CG 95°C, Azu-CG 95°C, aSP-CG 95°C.


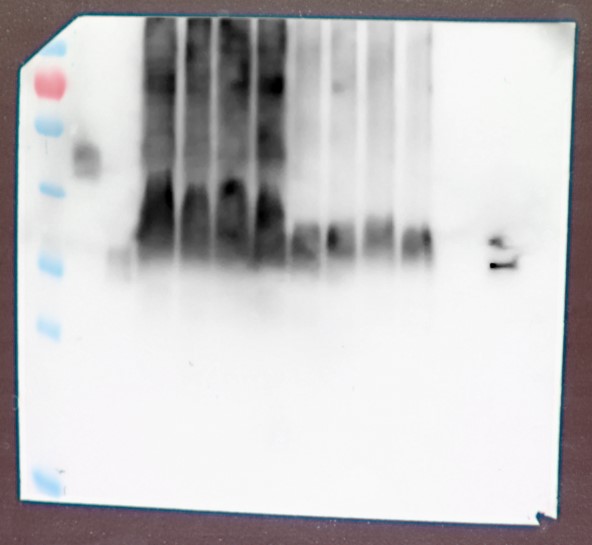


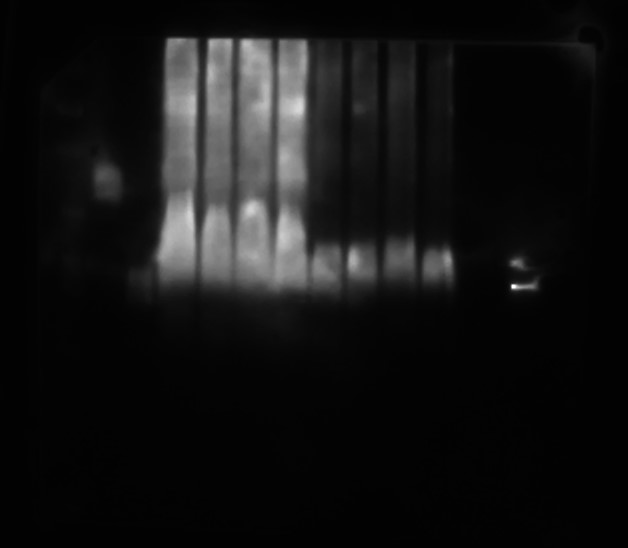


LH B-chain
Loading order: MW ladder, standard, standard 95°C, NSP-LH, HSA-LH, Azu-LH, aSP-LH, NSP-LH 95°C, HSA-LH 95°C, Azu-LH 95°C, aSP-LH 95°C, x, NSP-LH prec, HSA-LH prec, Azu-LH prec, aSP-LH prec.


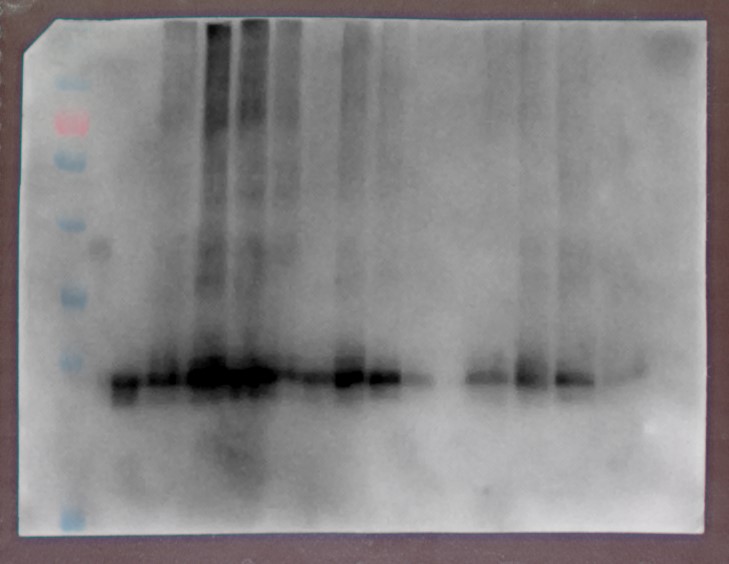


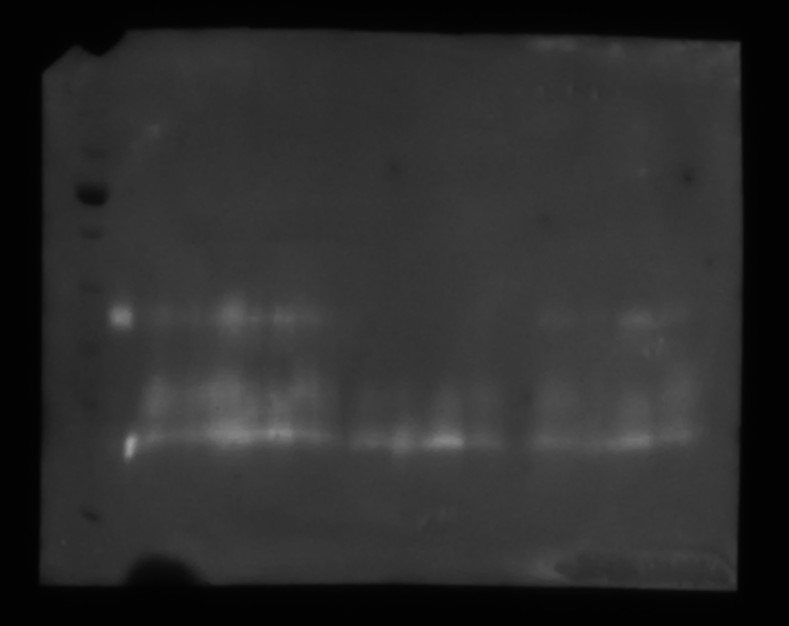


TSH B-chain
Loading order: MW ladder, —, NSP-TSH, HSA-TSH, Azu-TSH, aSP-TSH, —, NSP-TSH 95°C, HSA-TSH 95°C, Azu-TSH 95°C, aSP-TSH 95°C, —, HSA-TSH 95°C 30 ng, HSA-TSH 95°C 15 ng, HSA-TSH 95°C 7,5 ng. Heating the membrane after transfer for 20 min 95°C.


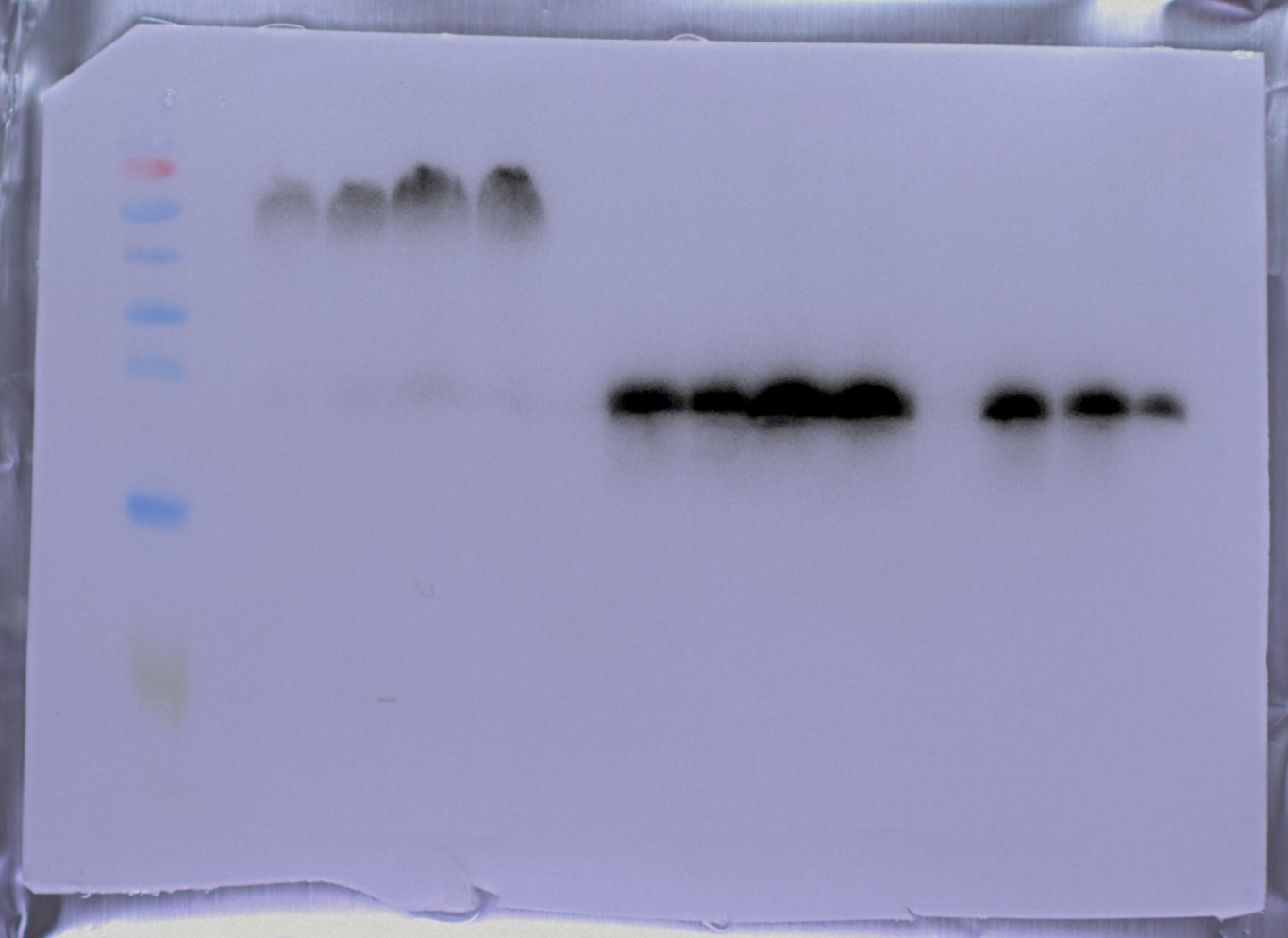


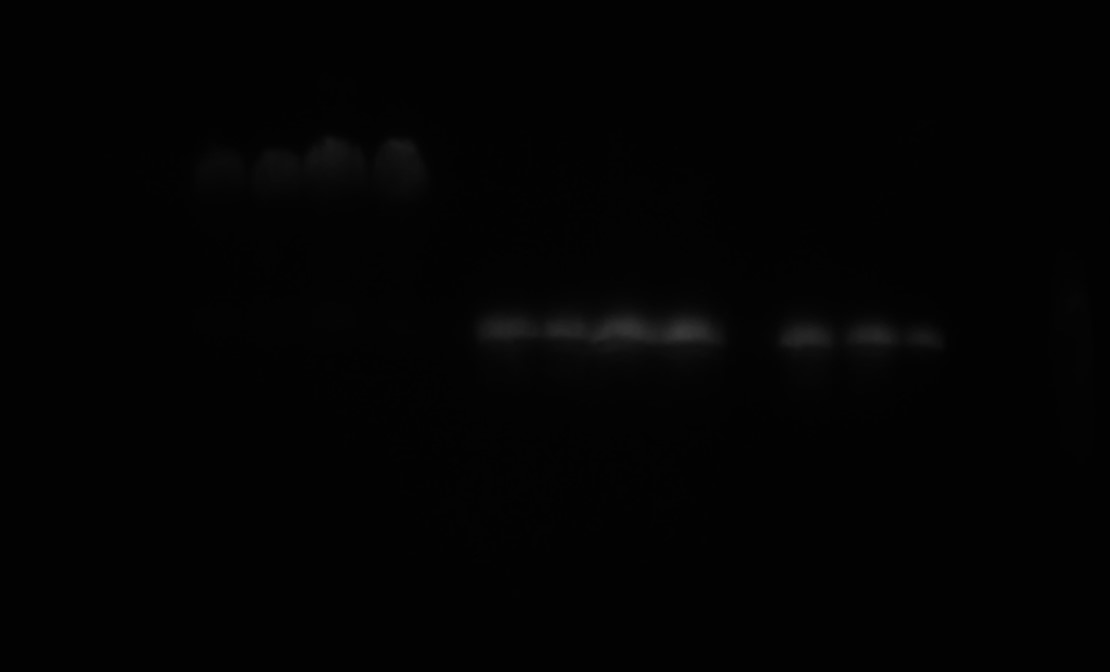


FSH B-chain
Loading order: MW ladder, standard, standard 95°C, NSP-FSH, HSA-FSH, Azu-FSH, aSP-FSH, NSP-FSH 95°C, HSA-FSH 95°C, Azu-FSH 95°C, aSP-FSH 95°C.


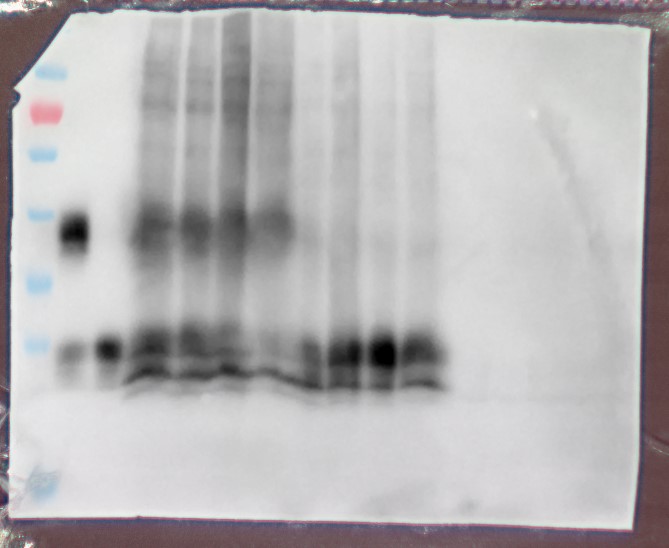


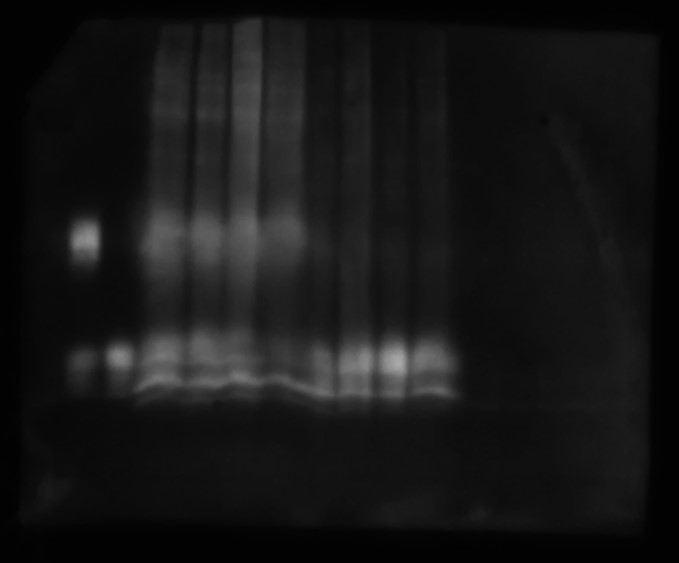


Method optimization for TSH-b staining (the sample HSA-TSH was used in all cases, the loading is indicated on the image):


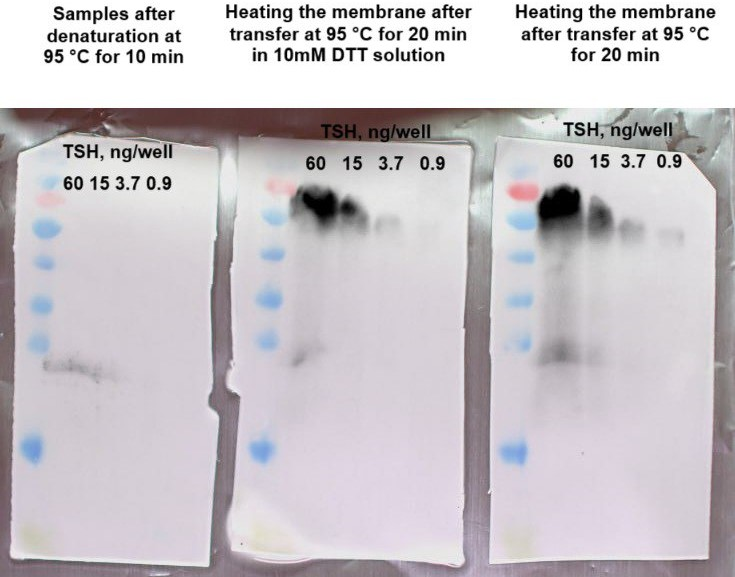


Legend:
The MW ladder – PageRuler Prestained Ladder (Thermo Scientific, #26616)
95°C – pretreatment by heating during 10 min at 95°C
prec – precipitated with ammonium sulfate
